# Gender differences in the microbial spectrum and antibiotic sensitivity of uropathogens isolated from patients with urinary stones

**DOI:** 10.1002/jcla.24155

**Published:** 2021-12-02

**Authors:** Jie Gu, Xiong Chen, Zhiming Yang, Yao Bai, Xiaobo Zhang

**Affiliations:** ^1^ Department of Geriatric Urology Xiangya International Medical Center Xiangya Hospital Central South University Changsha China; ^2^ National Clinical Research Center for Geriatric Disorders Xiangya Hospital Central South University Changsha China; ^3^ Martini‐Klinik Prostate Cancer Center University Hospital Hamburg‐Eppendorf Hamburg Germany; ^4^ Department of Urology Hunan Provincial People's Hospital The First Affiliated Hospital of Hunan Normal University Changsha China

**Keywords:** antibiotics susceptibility, bacterial spectrum, gender, urinary stones, urinary tract infection

## Abstract

**Purpose:**

The characteristics and resistance patterns of urine bacteriology urolithiasis patients between male and female have not been extensively studied. This study aims to investigate the gender differences in microbial spectrum and antibiotic susceptibility of uropathogens isolated from urolithiasis patients and provide insights for appropriate antimicrobial therapies.

**Materials and Methods:**

We retrospectively collected clinical microbiology data from urine culture in urolithiasis patients between March 2014 and December 2018 in Xiangya Hospital. Then the patients were divided into male and female groups. The microbial spectrum and frequency of susceptibility to antibiotics were compared.

**Results:**

A total of 359 uropathogen isolates were collected from 335 patients, including 144 males (43.0%) and 191 females (57.0%). *E. coli* dominated in both groups, indicating higher frequency in females (53.2%) than in males (26.6%, *p* < 0.001), followed by *E*. *faecalis*, with higher frequency in males (15.6%) than in females (2.9%, *p* < 0.001). Major Gram‐negative (*E. coli* and *K*. *pneumoniae*) bacteria showed high sensitivity to cefoperazone/sulbactam, cefotetan, piperacillin/ tazobactam, and amikacin. In contrast, the resistance level was high to penicillin, tetracycline, and vancomycin in both groups. Gram‐positive (*E*. *faecalis* and *E*. *faecium*) isolates demonstrated high sensitivity to gentamicin and vancomycin in both groups. Furthermore, uropathogens isolated from female urolithiasis patients were more susceptible to antimicrobials than males.

**Conclusions:**

Uropathogen microbial spectrum in female urolithiasis patients is different from males. High susceptibility antibiotics should be used empirically according to gender to avoid multidrug‐resistant bacteria increase.

## Graphical abstract

The uropathogen microbial spectrum in females with urinary stones is different from males. High susceptibility antibiotics should be empirically used according to gender to avoid increased multidrug‐resistant bacteria.

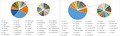

## 1 INTRODUCTION

Urolithiasis has a high prevalence and recurrence rate and is a significant public health issue with a high socioeconomic cost.^1^ Urinary calculi patients achieve a higher frequently associated with urinary tract infections (UTIs), which is a complication that precedes or follows urinary stone treatment.^2^, ^3^ Also, it causes a urinary stone formation, particularly *Proteus spp*, mainly formation of struvite crystals is associated with urease‐producing bacteria.^4^ Moreover, it is a source of recurrent UTIs, and stones may contain bacteria on their matrix or surface.^5^

Urinary calculi‐associated infections may cause severe complications due to endourological removal procedures, leading to renal insufficiency, urosepsis, and even life‐threatening.^6^ The high‐pressure irrigation system increases urinary tract pressure, causing the spread of bacteria. Therefore, it is essential to choose appropriate antibiotics to manage patients with symptomatic UTIs and prophylaxis prior to surgical procedures.

Studies showed that Gram‐negative bacteria Escherichia coli is the most frequent uropathogens.^3^, ^7^ Local antibiotic susceptibility and microbiological flora are extremely helpful planning adequate pre‐ and post‐operative empirical antibiotic therapy when microbiological assessment results are temporarily unavailable. However, urine bacteriology in patients with urinary stones appears to have a complex pattern.^8^, ^9^ It is unclear whether men and women with UTI stones have the same antimicrobial efficacy.

So far, there has not been extensive research on differences in urine bacteriology characteristics and susceptibility patterns between males and females with urinary stones. Therefore, we conducted this study to investigate the antimicrobial sensitivity of the main species isolates of bacteria associated with UTIs in urolithiasis patients and to get fundamental appropriate antimicrobial therapies.

## 2 MATERIALS AND METHODS

### 2.1 Patients

We retrospectively collected clinical microbiology data of positive midstream urine culture in urolithiasis patients from clinical laboratory between March 2014 and December 2018. Then the patients were divided into male and female groups. Non‐enhanced CT was employed to diagnose urinary calculi. Patients who prior used antibiotics within the last four weeks or with pregnancy, diabetes, chronic urine retention, neurogenic bladder, or immunosuppressive conditions were excluded. Urine was sampled before antibacterial treatment.

### 2.2 Urine culture

A clean midstream urine sample was routinely collected into a sterile container and sent to clinical for culture‐based microbiology. 10 μl of the urine sample with suspected bacterial infection was inoculated onto a blood agar plate and incubated at 37°C 18–24 h (cultured for seven days at 28°C with suspected fungal infection). If there is no bacterial growth, the culture will be extended to 48 h. Stainability and morphology of colonies were analyzed under a microscope and by mass spectrometry.

### 2.3 Drug sensitivity test

Furthermore, drug sensitivity and resistance testing were conducted using the microbroth dilution method. The antimicrobial susceptibility standards were followed when determining the pathogenic bacterial colonies MIC reference range. A sample was considered positive if a single micro‐organism was isolated with a concentration of >10^5^ CFU/ml and related to microscopy findings of >5 leucocytes per high power field.

The main reagents in the drug sensitivity test include yeast‐like fungal drug‐sensitive reaction strip (biomerieux Company) and a drug‐sensitive reaction card (biomerieux Company).^10^ A bacterial turbidimeter (biomerieux Company) and incubator (Hangzhou Lefeng Technology) were used as the drug sensitivity test equipment. All the data have been retrospectively collected from Department of Clinical Laboratory.

### 2.4 Statistical analysis

Continuous data were presented as means ± SDs. The chi‐square test and Mann–Whitney U test were employed to compare uropathogen distribution and susceptibility between two groups. Where a chi‐square test was not suitable, Fisher's exact probability test was used. *p* < 0.05 was considered statistically significant. SPSS version 21.0 software was utilized for data analysis.

## 3 RESULTS

### 3.1 Characteristics of the patients

**FIGURE 1:**
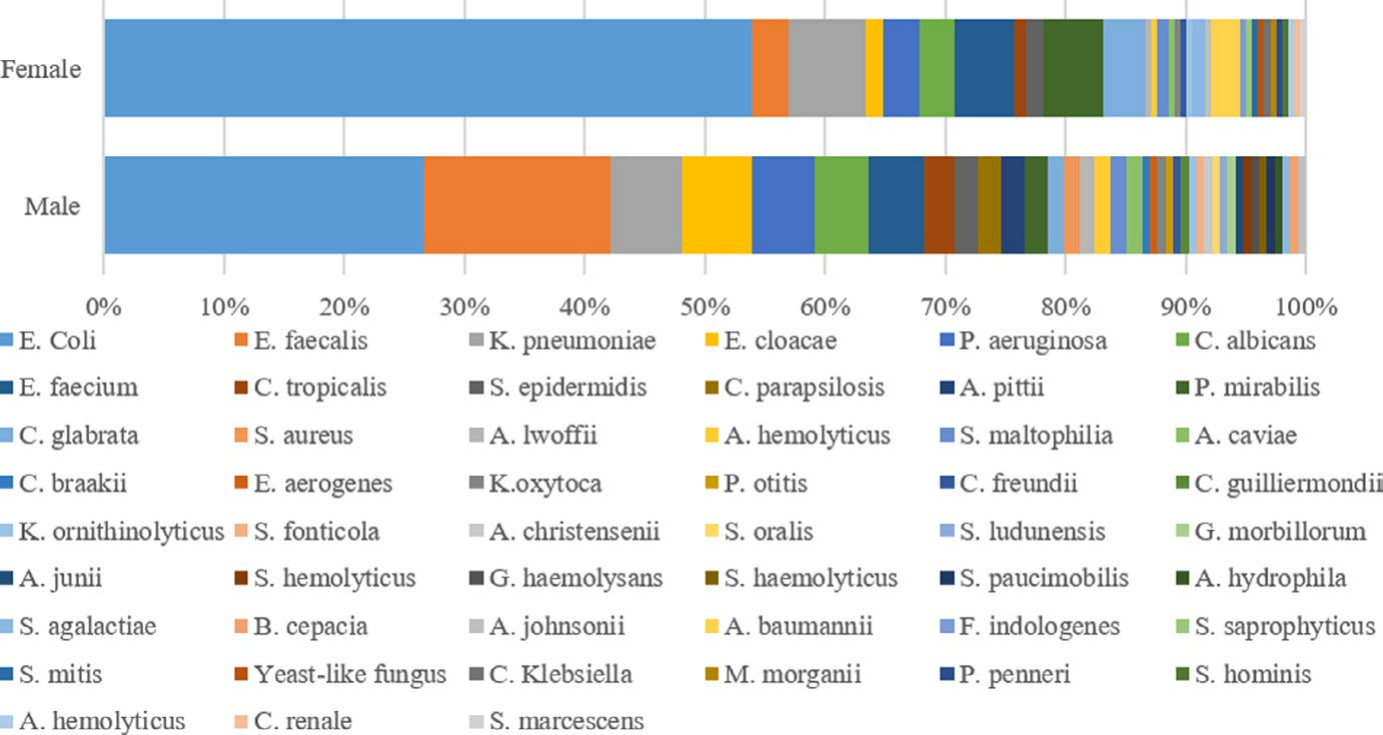
Microbial spectrum of uropathogens isolated from patients with urinary stones in male and female

A total of 359 uropathogens were detected in 335 patients with an average age of 53.56 ± 12.31 years old recruited from Xiangya hospital from March 2014 to December 2018 five consecutive years. Patients included 191 (57.0%) females (50.77 ± 12.23 years) and 144 (43.0%) males (54.1 ± 19.4 years). (Figure 1).

### 3.2 The bacterial spectrum isolated from male and female patients with urinary calculi

**FIGURE 2:**
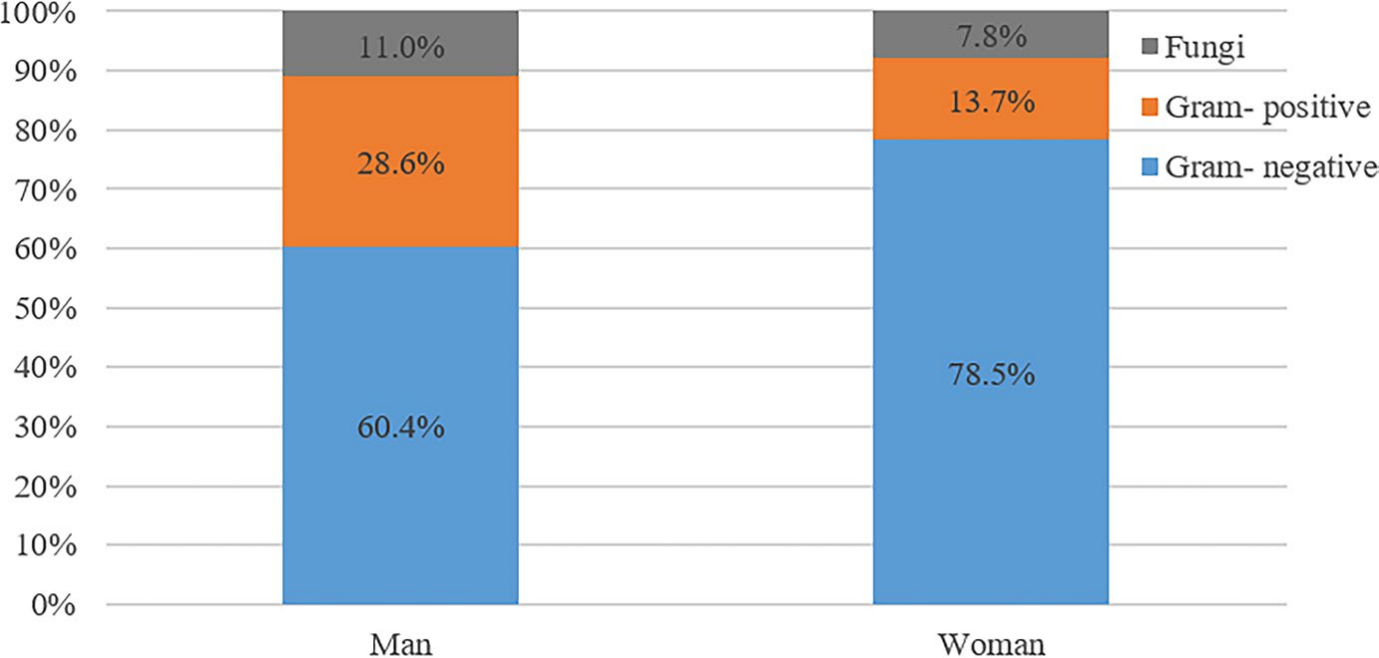
Detection rates of uropathogens in patients with stones between male and female

The bacterial spectrum between male and female patients is significantly different (Table 1 and Figure 1). Particularly, *E. coli* dominated in both males and females, and it was significantly more frequent in females (53.2%) than in males (26.6%, *p *< 0.001). *E*. *faecalis* was the second most common uropathogen inferior only to *E. coli*, with higher detected in males (15.6%) than in females (2.9%, *p *< 0.001). Furthermore, the incidence of *E*. *cloacae* infection was higher in males (5.8%) than in females (1.5%, *p *= 0.047). *P*. *mirabilis* infection, which mainly caused struvite formation, was higher in female patients (4.9%) than in male patients (1.9%). *E. coli* producing ESBL (+) was significantly higher in males (78.0%) than in females (53.2%, *p *= 0.006). As shown in Table 1, accompanied by urolithiasis in male and female patients, fourteen types of bacteria are responsible for the top 12 most frequently detected uropathogens, respectively, accounting for 78.6% and 82.3% of the total uropathogens. Thirty‐nine bacteria types were isolated from 154 males and 33 types from 205 females. Uropathogen detection rates between males and females are displayed in Figure 2. Gram‐negative bacteria dominated in both groups, with a higher rate in females (78.5%) than in males (60.4%, *p *< 0.001). In Gram‐positive bacteria, a higher rate was observed in males than females (28.6% vs. 13.7%, *p *< 0.001).

**TABLE 1:** Comparison of top 12 most frequently detected uropathogens in urinary stone patients between man and woman

Man	*N* (%) *N* = 154	*p* Value	Woman	*N* (%) *N* = 205	*p* Value
Escherichia coli^N^	41 (26.6)	0.000*	Escherichia coli^N^	109 (53.2)	0.000*
E. coli ESBL (+)	32 (78.0)	0.006*	E. coli ESBL (+)	58 (53.2)	0.006*
E. coli ESBL (−)	9 (22.0)		E. coli ESBL (−)	51 (46.8)	
Enterococcus faecalis^P^	24 (15.6)	0.000*	Klebsiella pneumoniae^P^	13 (6.3)	0.846
Klebsiella pneumoniae^N^	9 (5.8)	0.846	K. pneumoniae (+)	3 (23.1)	0.962
K. pneumoniae (+)	2 (22.2)	0.962	K. pneumoniae (−)	10 (76.9)	
K. pneumoniae (−)	7 (77.8)		Proteus mirabilis^N^	10 (4.9)	0.236
Enterobacter cloacae^N^	9 (5.8)	0.047*	Enterococcus faecium^P^	10 (4.9)	0.883
Pseudomonas aeruginosa^N^	8 (5.2)	0.272	Candida glabrata^F^	7 (3.4)	0.353
Candida albicans^F^	7 (4.5)	0.417	Candida albicans^F^	6 (2.9)	0.417
Enterococcus faecium^P^	7 (4.5)	0.883	Enterococcus faecalis^P^	6 (2.9)	0.000*
Candida tropicalis^F^	4 (2.6)	0.237	Pseudomonas aeruginosa^N^	6 (2.9)	0.272
S. epidermidis^P^	3 (1.9)	1	Acinetobacter baumannii^N^	5 (2.4)	0.073
Candida parapsilosis^F^	3 (1.9)	0.078	S. epidermidis^P^	3 (1.5)	1
Acinetobacter pittii^N^	3 (1.9)	0.078	Staphylococcus aureus^P^	3 (1.5)	1
Proteus mirabilis^N^	3 (1.9)	0.236	Enterobacter cloacae^N^	3 (1.5)	0.047*

Note

Chi‐square test was performed to detect differences in specific uropathogen distribution. Where a chi‐square test was not suitable, Fisher's exact probability test was used. *p < 0.05 illustrates statistical significance. ^N^Gram‐negative, ^P^Gram‐positive, ^F^Fungi.

### 3.3 The susceptibility of major gram‐negative bacteria (E. coli and K. pneumoniae) to antimicrobial drugs

*E. coli* demonstrated a higher susceptibility in females than males to ceftriaxone, cefazolin, ceftazidime, cefotetan, gentamicin, piperacillin/tazobactam, aztreonam, imipenem, compound trimethoprim, levofloxacin, amikacin, and ciprofloxacin (*p *< 0.05). *E. coli* bacteria showed more than 60% sensitivity in males and females to cefoperazone/sulbactam, cefotetan, piperacillin/tazobactam, nitrofurantoin, and amikacin, whereas the resistance level was high in both groups to penicillin, tetracycline, vancomycin, and ampicillin. Ceftriaxone and cefpodoxime presented a higher susceptibility to *K*. *pneumonia* in males than females (*p *< 0.05). *K*. *pneumonia* bacteria exhibited more than 60% sensitivity in males and females to cefotetan, piperacillin/tazobactam, imipenem, compound trimethoprim, and amikacin. In contrast, the resistance level was high in both groups to penicillin, tetracycline, vancomycin, ampicillin, and nitrofurantoin (Table 2). Higher susceptibility trends were observed in females than males (Mann–Whitney U test) regarding Major Gram‐negative bacteria E. coli (*p *< 0.001) and K. pneumoniae (*p *= 0.006).

**TABLE 2:** Susceptibility of main Gram‐negative bacteria to common antibiotics

Antibiotics	Escherichia coli			Klebsiella pneumoniae		
	Man	Woman	*p*	Man	Woman	*p*
Ceftriaxone	7.5%	39.4%	0.000*	22.2%	75.0%	0.030*
Cefazolin	5.0%	32.7%	0.001*	22.2%	66.7%	0.080
Cefpodoxime	20.0%	29.8%	0.236	66.7%	16.7%	0.032*
Ceftazidime	40.0%	68.3%	0.002*	44.4%	75.0%	0.203
Cefoperazone/Sulbactam	67.5%	79.8%	0.119	55.6%	83.3%	0.331
Cefotetan	67.5%	82.7%	0.047*	66.7%	83.3%	0.611
Penicillin	0.0%	1.0%	1.00	0.0%	0.0%	‐
Tetracycline	0.0%	0.0%	‐	0.0%	0.0%	‐
Gentamicin	35.0%	64.4%	0.001*	33.3%	75.0%	0.087
Tobramycin	45.0%	60.6%	0.091	33.3%	66.7%	0.198
Vancomycin	0.0%	1.0%	1.00	0.0%	0.0%	‐
Ampicillin	2.5%	13.5%	0.104	0.0%	0.0%	‐
Piperacillin/Tazobactam	62.5%	89.4%	0.000*	66.7%	75.0%	1.00
Aztreonam	27.5%	56.7%	0.002*	55.6%	75.0%	0.397
Imipenem	77.5%	91.3%	0.024*	77.8%	75.0%	1.00
Meropenem	22.5%	38.5%	0.070	11.1%	33.3%	0.338
Compound trimethoprim	25.0%	58.7%	0.000*	66.7%	66.7%	1.00
Nitrofurantoin	77.5%	76.9%	0.094	0.0%	8.3%	1.00
Levofloxacin	10.0%	41.3%	0.001*	33.3%	66.7%	0.198
Amikacin	77.5%	94.2%	0.003*	77.8%	83.3%	1.00
Ciprofloxacin	10.0%	40.4%	0.001*	33.3%	66.7%	0.198

*Statistically significant based on chi‐square test (*p*< 0.05). Where a chi‐square test was not suitable, Fisher's exact test was used.

### 3.4 The susceptibility of major gram‐positive bacteria (E. faecalis and E. faecium) to antibiotics

Table 3 shows susceptible rates of major Gram‐positive bacteria (*E*. *faecalis* and *E*. *faecium*) to various antibiotics. Overall, a higher susceptibility to ceftazidime and aztreonam was observed in both uropathogens (*p *< 0.05). Gentamicin, vancomycin, and nitrofurantoin revealed a high level in vitro susceptibility to both *E*. *faecalis* and *E*. *faecium*, whereas aminoglycosides and cephalosporins indicated relatively high resistance rates. Ceftazidime, piperacillin/tazobactam, and compound trimethoprim exhibited 40% susceptibility in females but <5% to *E*. *faecalis*. Penicillin, nitrofurantoin, and ciprofloxacin confirmed high susceptibility rate >60% to *E*. *faecalis*, and <20% susceptibility was observed to *E*. *faecium*.

**TABLE 3:** Susceptibility of main Gram‐positive bacteria to common antibacterial drugs

Antibiotics	Enterococcus faecalis			Enterococcus faecium		
	Man	Woman	*p*	Man	Woman	*p*
Ceftriaxone	0.0%	0.0%	‐	0.0%	0.0%	‐
Cefazolin	0.0%	0.0%	‐	0.0%	0.0%	‐
Ceftazidime	0.0%	40.0%	0.026*	0.0%	0.0%	‐
Cefoperazone/Sulbactam	17.4%	40.0%	0.285	0.0%	10.0%	1.00
Cefotetan	8.7%	40.0%	0.135	0.0%	0.0%	‐
Penicillin	60.9%	60.0%	1.00	20.0%	0.0%	0.333
Tetracycline	13.0%	0.0%	1.00	20.0%	20.0%	1.00
Gentamicin	60.9%	60.0%	1.00	80.0%	50.0%	0.580
Tobramycin	0.0%	20.0%	0.179	0.0%	0.0%	‐
Vancomycin	82.6%	60.0%	0.285	100.0%	100.0%	‐
Ampicillin	69.6%	80.0%	1.00	20.0%	10.0%	1.00
Piperacillin/Tazobactam	13.0%	40.0%	0.207	0.0%	0.0%	‐
Aztreonam	0.0%	40.0%	0.026*	0.0%	0.0%	‐
Imipenem	8.7%	40.0%	0.135	0.0%	0.0%	‐
Meropenem	4.3%	0.0%	1.00	0.0%	0.0%	‐
Compound trimethoprim	4.3%	40.0%	0.073	0.0%	0.0%	‐
Nitrofurantoin	60.9%	80.0%	0.626	20.0%	20.0%	1.00
Levofloxacin	73.9%	100.0%	0.553	20.0%	20.0%	1.00
Amikacin	13.0%	40.0%	0.207	0.0%	0.0%	‐
Ciprofloxacin	56.5%	60.0%	1.00	20.0%	10.0%	1.00

*Statistically significant based on chi‐square test (*p *< 0.05). Where a chi‐square test was not suitable, Fisher's exact probability test was used.

## 4 DISCUSSION

Herein, significant differences were observed in uropathogen microbial spectrum between male and female patients with urinary stones. E. coli was the most common bacteria in both groups, possessing higher frequency in females (53.2%) than in males (26.6%, *p *< 0.001). It may be explained by colonization of gastrointestinal pathogens around periurethral, since these bacteria are common flora in the gastrointestinal tract. Women have short urethral than men, this anatomical and physiological features increased the risk of bacterial rise from the perianal region into bladder.^11^ However, less than 62%–75% of studies reported uncomplicated UTIs.^12^, ^13^ This discrepancy might be due to more complex bacterial patterns in stone patients which were mainly reflected in the presence of calculi, invasive procedures, catheter‐associated placement, etc.^14^

It was notable that *E*. *faecalis* was the second most common uropathogen in patients with stones. In contrast to our findings, studies showed that *K*. *pneumonia* was the second frequent bacteria in hospital and community‐acquired UTIs.^12^, ^13^ It may due to the reason that *E*. *faecalis* was prevalent in catheter‐associated UTIs.^15^ Urinary catheters provide a surface for *E*. *faecalis* attachment and biofilm formation, promoting *E*. *faecalis* persistence in the bladder and further dissemination to the kidneys.^16^ Interestingly, *E*. *faecalis* was especially higher in males than females (15.6% vs. 2.9%, *p *< .001), possibly because elderly men typically suffer urodynamic dysfunction owing to prostatic hypertrophy, indwelling or intermittent bladder catheterization as the most effective and commonly used treatment.^17^, ^18^ What's more, several explanations have been suggested that males at the end of the lifespan exhibit an increased incidence of UTI. Uncircumcised was clearly a risk factor for UTI than their circumcised counterparts.^19^ In addition, various chronic conditions in males (diabetes and spinal cord injury) also promote UTI.^18^

According to EAU Urological Infections Guidelines 2020, the suggested first‐line treatment for empirical antimicrobial therapy in uncomplicated UTI was ciprofloxacin, levofloxacin, cefotaxime, and ceftriaxone. Cefepime, piperacillin/tazobactam, gentamicin, and amikacin are recommended as the second‐line treatment. Complicated UTI patients should be initially treated with an intravenous antimicrobial regimen such as aminoglycoside with or without amoxicillin, or a second or third‐generation cephalosporin, or extended‐spectrum penicillin with or without aminoglycoside.^20^ Drug selection should be based on local drug resistance research, and the protocol should be adjusted according to drug susceptibility.^21^

The antimicrobial sensitivity and resistance interpret that empirical antibiotic choice should take the patient gender into consideration. Overall, male's uropathogens in urolithiasis patients present a lower antibiotics sensitivity than those isolated from females. These differences were more apparent for main Gram‐negative bacteria *E. coli* and *K*. *pneumonia*, specifically for the antibiotics, including ceftazidime, aztreonam, compound trimethoprim, levofloxacin, and ciprofloxacin, which were more suitable to treat female UTI patients with urinary stones than male. For the major Gram‐positive bacteria *E*. *faecalis*, higher susceptibility to ceftazidime and aztreonam was observed in females than males (*p *< 0.05). Studies also found gender‐related differences in antimicrobial resistance. The fluoroquinolones were found unsuitable for the treatment of males. *E. coli* isolated from males showed resistance to the majority of antibiotics.^22^, ^23^

Finally, antibiotic benefits to patients are clear. However, their overuse and misuse have contributed to the growing problem of uropathogenic bacteria resistance, a serious threat to public health.^24^ Uropathogens isolated from patients with urinary stones manifested multidrug resistance in this study. Avoiding the rapid rise in antimicrobial resistance was a crucial and challenging task, and careful or appropriate antibiotic usage in all clinical situations is the fundamental resistance solution. To avoid inappropriate empiric antibiotic treatment, local microbial spectrum and antibiotic sensitivity of uropathogens isolated from patients with stones should be continuously studied and updated.

One of the study limitation is that the uropathogens isolated from upper and lower urinary stones were not distinguished and compared. Besides, it would have been more accurate if the results of stone culture had been combined (even blood culture if necessary). Also midstream urine cultures might not completely reflect UTI reality. Given the design of the study, which focused specifically on gender differences in cultured urinary bacteria for patients with urolithiasis, further studies are required in male and female controls with no history of stones or UTIs, and definitely, it would make our study more substantial. The data were collected from outpatient and inpatient department, and not all the patients have undergone surgery, so part of the stone analysis, stone features (Hounsfield Unit), urine pH, and catheter indwelling state were missing. Otherwise, subanalyses could be performed among those parameters to get more comprehensive results. Finally, this is a single‐center study, and further multi‐center and prospective studies are required.

## 5 CONCLUSIONS

The uropathogen microbial spectrum in females with urinary stones is different from males. High susceptibility antibiotics should be used empirically according to gender to avoid increased multidrug‐resistant bacteria.

## CONFLICT OF INTEREST

All authors declare no conflicts of interest in this study.

## AUTHOR CONTRIBUTIONS

All authors made contributions to the study, and Jie Gu performed database analysis and drafted the study. Xiaobo Zhang and Yao Bai contributed to all the designs of the study. Xiong Chen, Zhiming Yang, Yao Bai, and Xiaobo Zhang reviewed the draft of study. Jie Gu collected the data.

## Data Availability

The dataset generated during and/or analyzed during the current study is available from the corresponding author on reasonable request.
